# Multiple Dural Tuberculomas Presenting as Leptomeningeal Carcinomatosis

**DOI:** 10.1155/2011/581230

**Published:** 2011-07-13

**Authors:** Hasan Kocaeli, Özgür Taşkapılıoğlu, Elif Başaran, Ahmet Karaoğlu, Ahmet Bekar

**Affiliations:** Department of Neurosurgery, Uludağ University Medical Faculty, Görükle, 16059 Bursa, Turkey

## Abstract

*Objective and Importance*. We present the rare occurrence of multiple dural-based tuberculomas mimicking leptomeningeal carcinomatosis in a young immunocompetent patient. *Clinical Presentation*. A 36-year-old man presented with a 2-month history of generalized epileptic activity and altered perception. Neurological examination was remarkable for bilateral Babinski's sign. Cranial magnetic resonance imaging (MRI) revealed multiple dural-based enhancing lesions with cerebral edema. *Intervention*. A right frontal craniotomy was performed for diagnosis. Histological examination revealed multiple confluent necrotizing and nonnecrotizing granulomas with giant cells which was consistent with tuberculosis (TB), and the patient was placed on anti-TB therapy for 24 months. *Conclusion*. To the best of our knowledge isolated diffuse involvement of the dura mater by TB, mimicking leptomeningeal carcinomatosis, as the sole manifestation of disease has not been reported before. Since pachymeningeal TB is rarely suspected when atypical radiological appearance is combined with the absence of systemic disease, biopsy is inevitably required for diagnosis.

## 1. Introduction

Tuberculosis (TB) is a common central nervous system (CNS) infection that is becoming increasingly prevalent and is still a major cause of death or significant neurological disability [1]. Acquired immunodeficiency syndrome and the multidrug-resistant TB are the factors that have been reported to be associated with this increase [2]. With advanced means of disease detection and the availability of better antitubercular drugs, unusual forms of TB are increasingly encountered [3]. Chronic meningitis and intraparenchymal granulomatous lesions are the common presentations of CNS TB. Cases of isolated meningeal tuberculomas most of which resemble meningiomas have been reported in the presence of systemic disease [4–6]. To the best of our knowledge isolated diffuse involvement of the dura mater by TB, mimicking leptomeningeal carcinomatosis, as the sole manifestation of disease has not been reported before. We report a case that presented with multiple dural tuberculomas in the absence of systemic disease and highlight the difficulties that may be encountered in establishing a diagnosis.

## 2. Case Report

**Figure 1:**
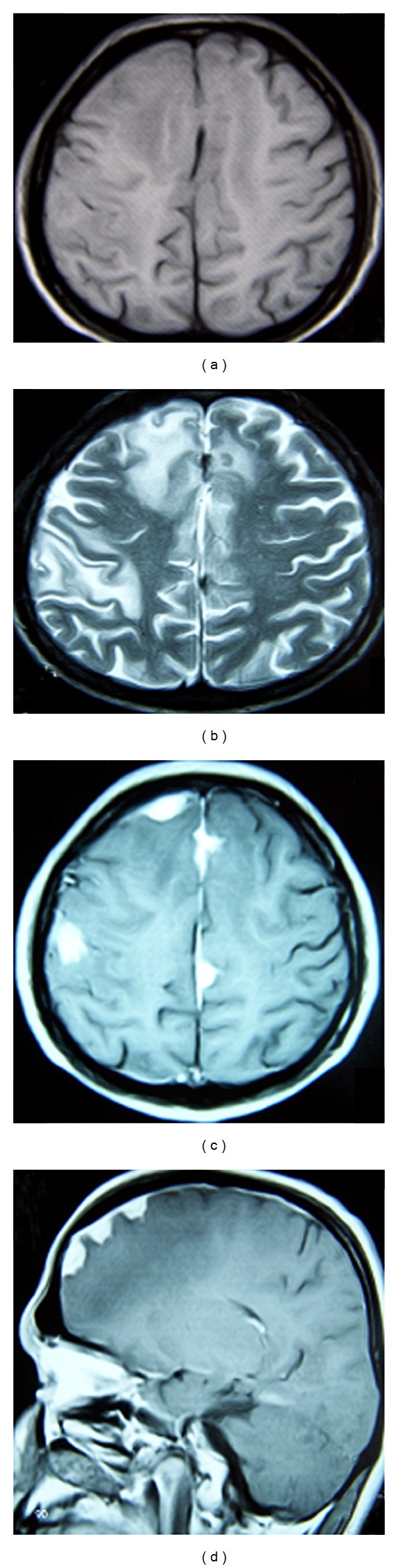
Cranial magnetic resonance imaging (MRI) shows multiple dural-based enhancing lesions on unenhanced (a) and enhanced T1-weighted axial (c) and sagittal (d) images with cerebral edema on T2-weighted images (b).

**Figure 2:**
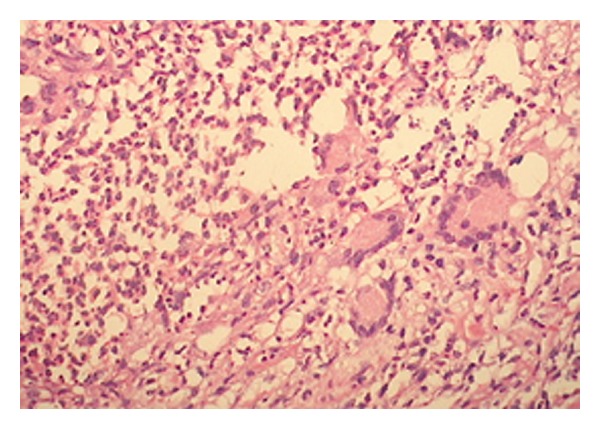
Histological examination revealed multiple confluent necrotizing and nonnecrotizing granulomas with giant cells (multinucleated and Langhans' type).

**Figure 3:**
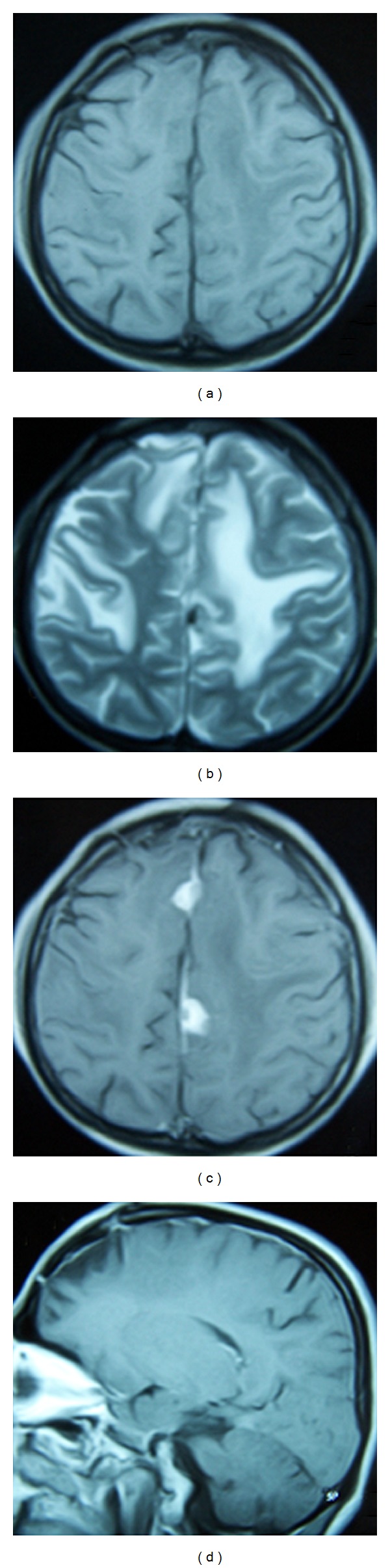
Follow-up MRI at the end of the second postoperative year shows regression of the lesions on unenhanced (a) and enhanced T1-weighted axial (c) and sagittal (d) images with cerebral edema on T2-weighted images (b).

This 36-year-old man presented with a 2-month history of generalized epileptic activity and altered perception. Upon examination by a neurologist he was begun on antiepileptic treatment, which consisted of carbamazepine 400 mg daily. His medical history was unyielding. Neurological examination was remarkable for bilateral Babinski's sign. Cranial magnetic resonance imaging (MRI) revealed multiple dural-based enhancing lesions with cerebral edema (Figure 1). Chest X-rays appeared normal, and the CSF was clear. The patient underwent a right frontal stereotaxic awake craniotomy for histopathological diagnosis. At craniotomy the outer layer of the dura was firm and thickened and avascular pinkish, firmly involved the inner layer, fibrous masses resembling meningioma. Histological examination revealed multiple confluent necrotizing and nonnecrotizing granulomas with giant cells (multinucleated and Langhans' type) (Figure 2). Microbiological studies did not reveal acid-fast bacilli. The patient was placed on a regimen of anti-TB medication for 18 months, which consisted of rifampicin (600 mg/daily), ethambutol (1500 mg/daily), and isoniazid (300 mg/daily). The patient's seizures were controlled with phenytoin 300 mg/day, and follow-up MRI at the end of the second postoperative year showed regression of the lesions (Figure 3).

## 3. Discussion

The global increase in incidence of TB is a health issue of universal concern. Most cases of CNS TB are caused by *Mycobacterium tuberculosis*. CNS involvement is believed to occur because of disease elsewhere in the body, mostly in the lungs. TB of the CNS becomes symptomatic in 10 to 20% of patients with extra pulmonary TB, and only 1% of patients develop intracranial tuberculoma. Intracranial tuberculoma usually results from the hematogeneous seeding of the tubercle bacilli to the leptomeninges and brain parenchyma, resulting in formation of tubercles, which enlarge, coalesce, and are walled by a fibrous capsule. The leptomeningeal tubercles either remain confined to the meninges forming fibrous masses attached to the dura, that is, tuberculoma en plaque or rupture into the subarachnoid space forming a Rich's foci [7]. The brain parenchyma surrounding the tuberculomas forms a thick fibrous capsule around the lesion, and they may enlarge sizably before the symptoms appear. The term “pachymeningeal TB” was suggested by Goyal et al. to describe cases of isolated dural-based lesions or lesions in which parenchymal involvement is believed to be caused by dural inflammation. Focal and diffuse patterns of tubercular pachymeningitis exist. Most of focal lesions of pachymeningeal tuberculosis are seen as en plaque, homogenous, uniformly enhancing, dural-based masses. The imaging findings of pachymeningeal TB are reported as follows: on plain computed tomography scans the lesions appear hyperdense while they appear isointense to brain parenchyma on T1-weighted MR images and isointense to hyperintense on T2-weighted images. In our case lesions appeared iso- and hypointense and hyperintense on T1-weighted and T2-weighted images, respectively.

The imaging findings of leptomeningeal involvement are not specific for TB, and a number of disease processes need to be considered in the differential diagnosis. These include leptomeningeal carcinomatosis, intracranial fibromatosis, lymphoma, meningioma, sarcoidosis, and syphilis [2]. Although various case reports of diffuse involvement of leptomeninges exist, multiple dural-based tuberculoma formation mimicking leptomeningeal carcinomatosis has not been reported before [8]. Anti-TB medications are the mainstay in the treatment of TB. Because the relapse rate is high after medication and the radiological disappearance of the lesions takes a long time, close followup with appropriate neuroimaging is critical, especially in immunocompromised patients. Poonnoose et al. conducted a study to determine the rate of radiological resolution of histologically proven tuberculomas treated with anti-TB therapy and found that more than two thirds of patients with partially excised or biopsied intracranial tuberculomas exhibited persistent lesions on CT scans even after 18 months after therapy. According to this data some patients with intracranial tuberculomas might require prolonged anti-TB therapy.

Since pachymeningeal TB is rarely suspected especially if there is atypical morphology and absent systemic disease, a biopsy is required to establish the diagnosis and institute specific treatment. However, craniotomy may be required in rare cases, which are refractory to medical treatment or if there is a significant mass effect.

## References

[1] Garcia-Monco JC (1999). Central nervous system tuberculosis. *Neurologic Clinics*.

[2] Bernaerts A, Vanhoenacker FM, Parizel PM (2003). Tuberculosis of the central nervous system: overview of neuroradiological findings. *European Radiology*.

[3] Goyal M, Sharma A, Mishra NK, Gaikwad SB, Sharma MC (1997). Imaging appearance of pachymeningeal tuberculosis. *American Journal of Roentgenology*.

[4] Bauer J, Johnson RF, Levy JM, Pojman DV, Ruge JR (1996). Tuberculoma presenting as an en plaque meningiomaz. *Journal of Neurosurgery*.

[5] Isenmann S, Zimmermann DR, Wichmann W, Moll C (1996). Tuberculoma mimicking meningioma of the falx cerebri. PCR diagnosis of mycobacterial DNA from formalin-fixed tissue. *Clinical Neuropathology*.

[6] Lindner A, Schneider C, Hofmann E, Soerensen N, Toyka KV (1995). Isolated meningeal tuberculoma mimicking meningioma: case report. *Surgical Neurology*.

[7] Dastur DK, Manghani DK, Udani PM (1995). Pathology and pathogenetic mechanisms in neurotuberculosis. *Radiologic Clinics of North America*.

[8] Dubey S, Devi BI, Jawalkar VK, Bhat DI (2002). Tuberculoma en plaque: a case report. *Neurology India*.

